# Formal Long-Term Care: Informal Caregivers’ Subjective Well-Being and Service Utilization

**DOI:** 10.5334/ijic.5565

**Published:** 2020-09-22

**Authors:** Wayne Freeman Weien Chong

**Affiliations:** 1 School of Social Sciences, Nanyang Technological University, SG; 2 GeroPsych Consultants Pte Ltd, SG

**Keywords:** informal caregiving, formal long-term care, service utilization, caregiver burden, caregiver depression, caregiver health status, integrated care

## Abstract

This thesis aimed to elucidate the role of informal caregiver subjective well-being in explaining formal long-term care service (LTCS) use. A systematic review and meta-analysis of literature found that elevated caregiver burden, caregiver depression, and poorer caregiver health status are associated with increased formal LTCS use. Quantitative analyses of longitudinal data collected from stroke survivors and their caregivers revealed that increased caregiving burden and caregiver depression are prospective and concurrent predictors of stroke rehabilitation use at 12-month post-stroke, and that non-distressed caregivers at 3-month post-stroke and 12-month post-stroke are likely to have cared for stroke rehabilitation users at 12-month post-stroke.

## Introduction

Lower than expected formal long-term healthcare service demand and unrelenting deleterious effects of informal caregiving on caregivers’ subjective well-being are evolving socioeconomic issues faced by many urban societies. These phenomena run counter to the needs of ageing populations, that presents challenges of a rise in chronic degenerative illnesses and long-term disabilities, and associated crowding at acute treatment facilities. The limited effectiveness of simply increasing formal long-term care service (LTCS) supply to meet these challenges has led to an expansion of demand-influencing strategies, which involve health and social care integration. This thesis aimed to elucidate the role of informal caregiver subjective well-being in explaining formal LTCS use.

Study 1 conceptualized informal caregiver subjective well-being as caregiver psychosocial needs, and asked if these needs are associated with formal LTCS use [1,2]. Study 2 measured caregiver well-being attributes, and examined if these attributes predict stroke rehabilitation use [3,4]. Study 3 used these attributes to identify caregiver psychosocial health latent profiles, and investigated if these profile transition patterns predict stroke rehabilitation use.

## Main Findings

Due to equivocal literature on the topic, a systematic review and meta-analysis of reported empirical data of moderate to high quality sampled from 31 journal articles and six theses was conducted. Study 1 found that research studies that involved fewer than 70% female informal caregivers reported 86% higher odds of caregivers experiencing higher depression levels associated with LTCS use by patients. Studies involving nursing homes reported 70% lower odds of caregivers experiencing higher burden associated with LTCS use. That only two non-USA studies contributed to the finding of 50% lower odds of caregivers experiencing poorer health status in relation to LTCS use suggest that a confirmation of this finding was required. That elevated burden and depression and poorer health status of the informal caregiver indicated a need for LTCS use beg an examination of their importance in influencing LTCS use in the presence of other known factors.

Using a longitudinal Singapore sample of stroke survivors and their primary informal caregivers, Study 2 showed that caregiver depression and caregiving burden were concurrent and prospective predictors of LTCS use, respectively. With other known factors controlled for, caregivers who experienced more burden at 3-month post-stroke, and those who were more depressed at 12-month post-stroke were respectively found to be 4% and 12% more likely to have cared for stroke rehabilitation users at 12-month post-stroke. Using the same sample, Study 3 showed that caregiver burden, depression and health status were useful indicators of psychosocial health latent profiles that differed regarding LTCS use. After controlling for other factors, non-distressed caregivers at 12-month post-stroke were found to have greater likelihood of having cared for stroke rehabilitation users at the same time point. Stroke rehabilitation users at 3-month post-stroke tended to continue using rehabilitation at 12-month post-stroke only when their caregivers were not distressed at 3-month post-stroke, but not when their caregivers were distressed. Distressed caregivers at baseline had a 24% probability of remaining distressed at 12-month post-stroke.

## Implications for Integrated Care Research and Practice

**Figure 1:**
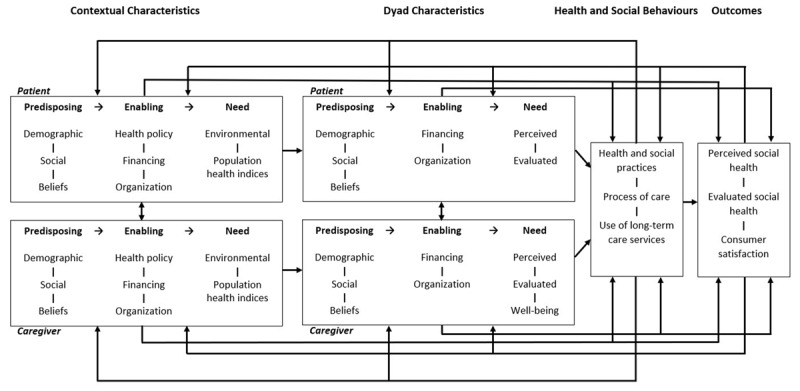
A Behavioural Model of Long-term Care Services Use.

This thesis shows that informal caregiver characteristics should be distinguished from patient characteristics, and that they individually and collectively explain LTCS use. It challenges assumptions of the established Behavioural Model of Health Services Use [5], that services use factors are individual characteristics and contexts. With primary and secondary evidence, this thesis argues for a caregiver dimension to be added alongside individual patient and contextual characteristics. Figure 1 shows a behavioural model of LTCS use proposed by this thesis. In this model, caregivers and patients operate in different contexts, although some contextual characteristics may be shared. With the emerging concept of health as “the ability to adapt and to self-manage” [6], separate but interacting caregiver and patient pathways should merge at LTCS use and other health and social behaviours of the dyad. Future research should test this model in various LTCS types and settings.

This thesis provides evidence that supports the premise that formal LTCS use decisions are made by the caregiver-patient dyad, rather than by the individual patient [7,8,9]. It challenges a key assumption of the dominant models of healthcare decision-making, that the individual patient is the sole unit of decision-making. Therefore, informal caregivers’ subjective well-being should be considered in integrated care service design and delivery. Caregiver needs may become more easily incorporated into care when community and family organizations and functions are merged with health care organizations and functions. Service integration could occur by first redefining service recipients to be the informal caregiver-patient dyad or the family. Then, multidisciplinary clinical teams could be equipped to manage the collective intentions and clinically relevant experiences of the dyad, on top of the presenting illness of the patient. Future research should investigate the experiences of the caregiver-patient dyad, and the mechanisms through which informal caregivers participate in decision-making in, and actual use of, formal LTCS.

This thesis suggests the possibility of a coexistence of positive and negative caregiving experiences by finding that caregiver depression was predictive of stroke rehabilitation use at 12-month post-stroke in Study 2, but that non-distressed caregivers were more likely to have cared for rehabilitation users at 12-month post-stroke in Study 3. A depressed caregiver may not experience distress due to resilience [10], effective coping strategies and presence of self-efficacy [11]. Caregiver resilience, which appears central to the emerging concept of health, is a potential outcome of integrated health and social care, and should be better understood through future research.

The probabilities of transition between distressed and non-distressed caregiver profiles over a 12-month duration found indicate the importance of integrating caregiver assessment early in the care continuum, such as during a patient’s hospital discharge planning and referral to community-based care. These transition probabilities also suggest the importance of incorporating and sustaining caregiver education and intervention as part of community-based care [12].

The results presented in this article are based on the author’s thesis presented at Nanyang Technological University, Singapore, on 4 May 2020. The full text is available from https://hdl.handle.net/10356/139476.

## Author Information

Dr. Wayne Chong is a non-executive director and consultant geropsychologist with GeroPsych Consultants Pte Ltd, a Singapore-based consultancy in which he specializes in evaluating long-term care services, and translating scientific knowledge into tools for seniors, caregivers, aged care providers and policy makers. Wayne is also an adjunct lecturer with the School of Social Sciences at Nanyang Technological University (NTU), Singapore. He holds a Ph.D. from NTU Singapore, and a PDClinPsyc from James Cook University. An award-winning conference presenter, Wayne has published and peer-reviewed manuscripts in local and international scientific journals in ageing and integrated care. He is now involved in transnational research that seeks to understand the impact of the Covid-19 pandemic on older adults and their integrated caregivers.
